# Neonatal outcomes and congenital malformations in children born after progestin-primed ovarian stimulation protocol

**DOI:** 10.3389/fendo.2022.965863

**Published:** 2022-11-09

**Authors:** Danjun Li, Zhijie Hu, Qiuju Chen, Weiran Chai, Renfei Cai, Yanping Kuang, Xuefeng Lu

**Affiliations:** Department of Assisted Reproduction, Shanghai Ninth People’s Hospital, Shanghai Jiaotong University School of Medicine, Shanghai, China

**Keywords:** progestin-primed ovarian stimulation, *in vitro* fertilization, live birth, congenital malformation, neonatal outcome

## Abstract

**Purpose:**

The purpose of this study is to assess the safety of progestin-primed ovarian stimulation (PPOS) protocol regarding the neonatal outcomes and congenital malformations in babies born after *in vitro* fertilization (IVF) and frozen embryo transfer (FET).

**Methods:**

In this large retrospective cohort study, a total of 16,493 infants born between 1 September 2013 and 31 July 2021 from IVF and FET cycles after treatment with either PPOS (n = 15,245) or gonadotropin-releasing hormone antagonist (GnRH-ant) (n = 1,248) were finally enrolled. The primary outcome measure was the incidence of congenital malformations. The secondary outcome measures were rates of low birth weight (LBW), very low birth weight (VLBW), preterm birth (PTB), very preterm birth (VPTB), and early neonatal death.

**Results:**

Birth characteristics for both singletons and twins regarding the sex of infants, gestational age, birth weight, and birth length were comparable between the PPOS group and the GnRH-ant group. Rates of LBW, VLBW, PTB, VPTB, and early neonatal death were also similar. The reanalysis using propensity score matching (PSM) and multivariable logistic regression indicated that the PPOS protocol could not increase the risk of adverse neonatal outcomes compared with the GnRH-ant protocol. Furthermore, no significant difference was observed in the overall incidence of congenital malformations in live-born babies. After PSM and controlling for all confounders, the results remained insignificant with an adjusted odds ratio of 0.66 [95% confidence interval (CI) 0.32–1.34] and 2.43 [95% CI 0.97–6.06], respectively, for singletons and twins.

**Conclusions:**

Our study suggests that compared with GnRH-ant treatment for IVF, the PPOS protocol could not produce a negative effect on the newborn population in terms of neonatal outcomes and congenital malformations.

## Introduction

With the wide spread of assisted reproductive technology (ART), an increasing number of infertile couples have successfully conceived *via in vitro* fertilization/intracytoplasmic sperm injection (IVF/ICSI) over the past decades. Currently, at least five million of infants have been born as a result of ART, approximately accounting for 2% of all births in European countries (1). Notably, this proportion may be gradually increasing each year. However, the potential risk of fetal adverse outcomes due to ART is always the source of debate. Several studies have reported the increasing risk of major congenital malformations of live-born babies following ART compared with natural conceptions (2–4). The increased incidence of birth defects in offspring might be mainly attributable to the infertility characteristics, ovarian stimulation regimens, or specific ART procedures (5, 6).

Controlled ovarian stimulation (COS) has always been considered to play a critical role in the development of ART. The application of exogenous gonadotropin resulting in supraphysical serum estradiol levels and the subsequently positive feedback at the pituitary body might hence cause a premature luteinizing hormone (LH) and premature luteinizaton (7). Consequently, in order to minimize the occurrence of premature LH surge, the coadministration of gonadotropin-releasing hormone agonists (GnRHa) or gonadotropin-releasing hormone antagonists (GnRH-ant) was applied into stimulation regimens and gradually accepted as routine regimens safe for maternal and neonatal health (8, 9). However, these solutions could be accompanied by the complication of ovarian hyperstimulation syndrome (OHSS) triggered by human chorionic gonadotropin (hCG) or complex stimulation (10).

Previously, studies have found that progestin (P) secreted during the luteal phase could strongly inhibit LH secretion and estradiol-induced positive feedback effects with no spontaneous LH surge (11). Moreover, considering the need for new methods with improved efficacy and safety, our center in 2015 firstly proposed to introduce oral P into the regimen for COS, namely progestin-primed ovarian stimulation (PPOS) (12, 13). P’s LH-suppression effects and popularized freeze-all protocol’s efficacy suggest that the PPOS protocol has the advantages of control over LH surge and OHSS incidence. The general concern regarding whether P used in COS is safe for the offspring has still not been resolved, and information about the safety of PPOS for live-born infants compared with GnRH analogues was limited. Prior studies have demonstrated that the neonatal outcomes and the risk of congenital malformations were comparable with the use of P, including medroxyprogesterone acetate (MPA), utrogestan, or dydrogesterone for the PPOS protocol when compared with the GnRHa short protocol (14–17), and a meta-analysis has concluded that the PPOS protocol, compared with the GnRHa protocol, was associated with a similar congenital malformation risk profile (18).

As one of routine COS procedures, the GnRH-ant protocol could reduce the incidence of OHSS without affecting the pregnancy and neonatal outcomes compared with the GnRHa protocol (19, 20). However, no comparison of neonatal outcomes has been made between the PPOS protocol and the GnRH-ant protocol. Moreover, to date, more than 10,000 infants have been born with the use of the PPOS regimen in our center. Therefore, a larger cohort retrospective study is needed to further assess the safety for the new COS regimen. Considering these, we chose the GnRH-ant protocol as the control group to comprehensively assess the neonatal outcomes and congenital malformations for the newborn population after PPOS in a larger sample.

## Materials and methods

### Study design and participants

This retrospective cohort study was performed in the Department of Assisted Reproduction of the Ninth People’s Hospital of Shanghai Jiao Tong University. The study protocol was approved by the institutional ethics committee of the Ninth Hospital. All participants gave written informed consent after describing the research in detail.

All infertile patients (n = 19,385) who underwent IVF/ICSI followed by frozen-thawed embryo transfer (FET) using the PPOS or GnRH-ant protocol were recruited for the study during the period between 1 January 2013 and 31 October 2020 in our center. Considering that these factors could be associated with birth defects, women with reported pregnancy-related diseases such as hypertension (n = 522), gestational diabetes mellitus (n = 1,321), thyroid diseases (n = 35), intrahepatic cholestasis of pregnancy (n = 59), anemia (n = 58), cardiac diseases (n = 13), and inflammatory diseases in pregnancy (n = 32) and combined disorders (n = 172) were excluded (21). Furthermore, cycles receiving donor sperm due to the possible consequence on neonatal outcomes (n = 194) were excluded (22). Cycles with missing core data (n = 245) were also excluded. A total of 16,493 infants born between 1 September 2013 and 31 July 2021 from IVF and FET cycles after treatment with either PPOS (n = 15,245) or gonadotropin-releasing hormone antagonist (GnRH-ant) (n = 1,248) were finally enrolled in this study. The flow diagram of the study design and cohort selection is shown in **Supplemental Figure 1**.

### Regimens

In the PPOS group, patients underwent daily intramuscular injection of 150 or 225 IU of human menopausal gonadotropin (hMG; Anhui Fengyuan Pharmaceutical Co., Ltd., Hefei, China) and simultaneously administered with 10 mg of MPA or 100 mg of utrogestan (Laboratories Besins International, Paris, France) or 20 mg of dydrogesterone (Duphaston, Abbott Biologicals, USA) beginning from the menstrual cycle day 2 or 3 (MC2 or 3) until the trigger day. In the GnRH-ant group, patients were injected daily with 150 or 225 IU hMG intramuscularly from MC2 or 3 onward and 0.25 mg of GnRH-ant (cetrotide; Baxter) from MC5 onward. Ovarian response was assessed according to the ultrasound monitoring and serum estradiol analysis. When the diameter of one dominant follicle reached 20 mm or at least three follicles reached diameters of 18 mm, the final oocyte maturation was triggered by 0.1 mg of triptorelin (decapeptyl, Ferring Pharmaceuticals) alone or co-triggered with 1,000–5,000 IU of hCG (Lizhu Pharmaceutical Trading Co., China).

All follicles with diameters of over 10 mm were retrieved within 32–36 h following maturation induction under transvaginal ultrasound guidance (based on per-group protocol). Then, the retrieved oocytes were fertilized *in vitro* by conventional IVF and/or ICSI, depending on the semen parameters. The embryos were further cultured in the Continuous Single Culture (CSC; Irvine Scientific, CA) supplemented with 10% serum substitute supplements (SSS; Irvine Scientific, CA). The cleavage-stage embryos (day 3) were graded according to the Cummins criteria (23). Grade I and II embryos regarded as top-quality were frozen by vitrification, and the remaining embryos (grades III and IV) were further cultured until the blastocyst stage. Thereafter, only blastocysts with good morphology were frozen. The freezing and thawing procedures were carried out as previously described (24).

Before embryos were thawed on the transfer of day, endometrial preparation for frozen embryo transfer (FET) could be performed in the natural cycle, artificial cycle, or mild stimulation cycle. The choice of method of endometrial preparation depends on the maternal infertile characteristics such as menstrual regularity. Then, no more than two thawed embryos were transferred for each patient. Once the pregnancy was achieved, P supplement would be continued until 10 weeks of gestations.

### Follow-up of pregnancy and neonatal outcomes

All patients who obtained clinical pregnancy were followed up in the form of a telephone interview and recorded every stage of pregnancy until 1 week after delivery. Information including pregnancy-related complications, infant gender, mode of delivery, birth weight and length, birth date and locality, gestational weeks, neonatal diseases, and presence of congenital malformations was included in the standardized questionnaires. Furthermore, detailed reports on pregnancies, deliveries, and neonatal outcomes could be obtained from the gynecologists and pediatricians in charge. For live-born babies with birth defects, case information was gathered by a specially designated nurse to make it clear whether the infants met the definition of the Chinese Birth Defects Monitoring Program.

### Outcome measures

The primary outcome of this study was the incidence of congenital malformations. The secondary outcome measures included low birth weight (LBW), very low birth weight (VLBW), preterm birth (PTB), very preterm birth (VPTB), and early neonatal death rates.

Congenital malformations were defined as the structural and functional anomalies as well as genetic defects occurring during pregnancy or at birth, which were classified based on the International Classification of Diseases Q Codes, 10th revision (ICD10: Q00-Q99) (25, 26). LBW and VLBW were considered as birth weight <2,500 and <1,500 g, respectively. PTB and VPTB were considered as delivery taking place before 37 and 32 completed weeks of gestation, respectively. Early neonatal death referred to the death of a live-born baby within 7 days of birth.

### Statistical analysis

Sample size calculation was not performed as this was an exploratory retrospective study, and there were no prior data to guide its sample size calculation. Statistical analyses were performed using Statistical Package for Social Sciences (version 25.0; SPSS Inc., USA) and R statistical programming language (version 4.1.0; R Foundation for Statistical Computing, Austria). The normality of continuous variables was tested by the histograms and Q–Q plots as well as the Kolmogorov–Smirnov test. Data with normal distribution were presented as the mean ± standard deviation (SD) or as medians for non-normal distribution, and between-group differences were compared *via* Student’s t-test or the Mann–Whitney U test, as appropriate, whereas categorical variables were described as number (percentage) and were compared *via* the chi-squared test or Fisher’s exact test when appropriate. Statistical significance was defined as a P value <0.05.

To balance maternal baseline characteristics between two groups, we established a one-to-one propensity score matching (PSM) model using the nearest-neighbor matching algorithm, and after matching, the balance between the two groups was evaluated by the standardized mean difference (<0.1). The potential confounding factors chosen for matching included maternal age, body mass index (BMI), duration of infertility, obstetrical history, cause of infertility, sperm origin, fertilization method, FET endometrial preparation, endometrial thickness, number of transferrable embryos, and embryo stage at transfer. Then, the multivariable logistic regression analysis was further performed to evaluate the possible association between the ovarian stimulation protocol (PPOS *vs*. GnRH-ant) and LBW, PTB, and congenital malformations after adjusting for the aforementioned confounders. The crude and adjusted odds ratios (ORs) with corresponding 95% confidence intervals (CIs) were calculated.

## Results

Originally, a total of 19,385 clinical pregnancy cycles were selected from our database. According to the exclusive criteria mentioned above, 16,734 clinical pregnancy cycles were ultimately recruited for our study including 15,382 cycles from the PPOS protocol and 1,352 cycles from the GnRH-ant protocol. For the treatment with the PPOS protocol, 12,988 ongoing pregnancies could lead to the birth of 15,245 live-born infants (12,317 live-birth cycles). Moreover, for the treatment with the GnRH-ant protocol, 1,092 ongoing pregnancies could lead to the birth of 1,248 live-born infants (1,039 live birth cycles). In addition, the selective terminations of pregnancy due to fetal anomalies are listed in **Supplemental Table 1**. We could see a comparable prevalence of congenital malformations in fetuses between the two groups.

As shown in **Table 1**, before matching, some maternal and cyclic parameters, such as maternal age, BMI, the proportion of primary infertility and nulliparity, sperm origin, and embryo stage at transfer, were comparable between the two groups. However, women in the GnRH-ant group tended to undergo a notably longer duration of infertility compared with women in the PPOS group (3.28 ± 2.61 *vs*. 3.01 ± 2.61, P < 0.001). Moreover, the cause of infertility for women in the GnRH-ant group might have significantly contributed more to male factors, endometriosis, and unexplained factor than women in the PPOS group (P = 0.001 <0.05). Furthermore, IVF was applied more frequently in women from the PPOS group than that from the GnRH-ant group (59.0% *vs*. 57.1%, P = 0.004 <0.05), whereas hormone replacement therapy was more preferably used for FET endometrial preparation in women from the GnRH-ant group than that from the PPOS group (37.8% *vs*. 31.1%, P < 0.001). Moreover, a significantly fewer proportion of double embryos was transferred in the PPOS group compared with the GnRH-ant group (81.7% *vs*. 84.4%, P = 0.028 <0.05). Regarding the thickness of the endometrium, it was significantly greater in the GnRH-ant group than in the PPOS group (10.91 ± 2.35 *vs*. 10.64 ± 2.17, P = 0.002 <0.05). After matching, all maternal baseline characteristics of 1,038 women per group were similarly adjusted, and no significant difference was observed between the two groups concerning all confounding factors.

**Table 1:** Maternal and cyclic characteristics of the live birth cycles between the PPOS and GnRH-ant groups.

	Before matching			After matching		
	PPOS (*n* = 12,317)	GnRH-ant (*n* = 1,039)	*P*-value	PPOS (*n* = 1,038)	GnRH-ant (*n* = 1,038)	*P*-value
Maternal age (years)	31.61 ± 4.05	31.84 ± 4.17	0.084	31.82 ± 4.03	31.84 ± 4.17	0.945
Maternal BMI (kg/m^2^)	21.49 ± 3.44	21.40 ± 3.73	0.436	21.50 ± 3.57	21.40 ± 3.73	0.529
Duration of infertility (years)	3.01 ± 2.61	3.28 ± 2.61	<0.001	3.20 ± 2.66	3.28 ± 2.61	0.521
Primary infertility, *n* (%)	7,067 (57.4)	613 (59.0)	0.309	604 (58.2)	612 (59.0)	0.755
Nulliparity, *n* (%)	11,331 (92.0)	956 (92.0)	0.985	949 (91.4)	955 (92.0)	0.691
Cause of infertility, *n* (%)			0.001			0.145
Male factor	1,221 (9.9)	134 (12.9)		114 (11.0)	133 (12.8)	
Tubal factor	4,761 (38.7)	377 (36.3)		411 (39.6)	377 (36.3)	
Endometriosis	200 (1.6)	24 (2.3)		21 (2.0)	24 (2.3)	
Unexplained	312 (2.5)	38 (3.7)		23 (2.2)	38 (3.7)	
Combined	5,823 (47.3)	466 (44.9)		469 (45.2)	466 (44.9)	
Sperm origin, *n* (%)			0.345			0.529
Ejaculated	12,024 (97.6)	1,007 (96.9)		1,002 (96.5)	1,007 (97.0)	
Testicular	245 (2.0)	26 (2.5)		32 (3.1)	25 (2.4)	
Epididymal	48 (0.4)	6 (0.6)		4 (0.4)	6 (0.6)	
Fertilization method, *n* (%)			0.004			0.059
IVF	7,262 (59.0)	593 (57.1)		637 (61.4)	593 (57.1)	
ICSI	3,361 (27.3)	328 (31.6)		278 (26.8)	327 (31.5)	
IVF+ICSI	1,694 (13.8)	118 (11.4)		123 (11.8)	118 (11.4)	
FET endometrial preparation, *n* (%)			<0.001			0.407
Natural cycle	2,546 (20.7)	186 (17.9)		169 (16.3)	186 (17.9)	
Mild stimulation	5,946 (48.3)	460 (44.3)		488 (47.0)	460 (44.3)	
Hormone replacement therapy	3,825 (31.1)	393 (37.8)		381 (36.7)	392 (37.8)	
Endometrial thickness (mm)	10.64 ± 2.17	10.91 ± 2.35	0.002	10.96 ± 2.31	10.90 ± 2.34	0.558
No. of embryos transferred, *n* (%)			0.028			0.314
Single	2,258 (18.3)	162 (15.6)		180 (17.3)	162 (15.6)	
Double	10,059 (81.7)	877 (84.4)		858 (82.7)	876 (84.4)	
Embryo stage transferred, *n* (%)			0.835			0.531
Cleavage stage	10,179 (82.6)	856 (82.4)		844 (81.3)	856 (82.5)	
Blastocyst stage	2,138 (17.4)	183 (17.6)		194 (18.7)	182 (17.5)	

Data are given as mean ± SD for continuous variables and n (%) for dichotomous variables. All P values were assessed with the use of χ^2^ or Fisher’s exact test (dichotomous variables) and t test or the Mann–Whitney U test (continuous variables).

PPOS, progestin-primed ovarian stimulation; GnRH-ant, gonadotropin-releasing hormone antagonist; BMI, body mass index; IVF, *in vitro* fertilization; ICSI, intracytoplasmic sperm injection; FET, frozen embryo transfer.

**Table 2** presents the neonatal outcomes of live-born singletons and twins before and after the analysis of PSM. As the results show, no significant differences regarding the sex of infants, gestational age, birth weight, and birth length were observed between the GnRH-ant and PPOS groups in both singletons and twins. Moreover, the overall incidence of early neonatal death was comparable between the two groups among singletons and twins. Singletons and twins born after treatment with PPOS exhibited similar rates of LBT, VLBT, PTB, and VPTB compared with that born after treatment with GnRH-ant.

**Table 2:** Neonatal outcome of live-born singletons and twins between the PPOS and GnRH-ant groups.

	Before matching			After matching		
	PPOS(*n* = 12,317)	GnRH-ant(*n* = 1,039)	*P*-value	PPOS(*n* = 1,038)	GnRH-ant(*n* = 1,038)	*P*-value
**Singletons**	9,389	830		776	829	
Sex of infants, *n* (%)			0.423			0.203
Male	4,900 (52.2)	437 (52.7)		384 (49.5)	437 (52.7)	
Female	4,486 (47.8)	392 (47.2)		392 (50.5)	391 (47.2)	
Unknown	3 (0)	1 (0.1)		0 (0)	1 (0.1)	
Birth weight (g)	3,334.7 ± 527.5	3,370.7 ± 1153.7	0.099	3,330 ± 546.5	3,370.5 ± 1154.4	0.773
Very low birth weight (<1,500), *n* (%)	42 (0.4)	5 (0.6)	0.587	3 (0.4)	5 (0.6)	0.727
Low birth weight (<2,500), n (%)	304 (3.2)	21 (2.5)	0.265	24 (3.1)	21 (2.5)	0.497
Gestational age (week)	39.0 ± 1.6	39.0 ± 1.5	0.196	39.0 ± 1.6	39.0 ± 1.5	0.196
Very preterm birth (<32), *n* (%)	72 (0.8)	5 (0.6)	0.599	4 (0.5)	5 (0.6)	1.000
Preterm birth (<37), *n* (%)	421 (4.5)	27 (3.3)	0.097	39 (5.0)	27 (3.3)	0.075
Birth length (cm)	50.8 ± 22.4	50.5 ± 18.6	0.775	50.6 ± 23.5	50.5 ± 18.6	0.968
Early neonatal death, *n* (%)	11 (0.1)	1 (0.1)	1.000	1 (0.1)	1 (0.1)	1.000
**Twins**	5,856	418		524	418	
Sex of infants, *n* (%)			0.776			0.758
Male	3,068 (52.4)	222 (53.1)		273 (52.1)	222 (53.1)	
Female	2,788 (47.6)	196 (46.9)		251 (47.9)	196 (46.9)	
Unknown	0 (0)	0 (0)		0 (0)	0 (0)	
Birth weight (g)	2,527.9 ± 461.7	2,508.7 ± 462	0.411	2,543.7 ± 418.9	2,508.7 ± 462	0.491
Very low birth weight (<1,500), *n* (%)	191 (3.3)	14 (3.3)	0.922	8 (1.5)	14 (3.3)	0.066
Low birth weight (<2,500), n (%)	2,274 (38.8)	170 (40.7)	0.457	201 (38.3)	170 (40.6)	0.471
Gestational age (week)	36.3 ± 2.1	36.2 ± 2.1	0.400	36.4 ± 1.7	36.1 ± 2.0	0.198
Very preterm birth (<32), *n* (%)	248 (4.2)	16 (3.8)	0.689	10 (1.9)	16 (3.8)	0.074
Preterm birth (<37), *n* (%)	2,658 (45.4)	208 (49.8)	0.083	240 (45.8)	208 (49.7)	0.227
Birth length (cm)	47.7 ± 3.2	47.7 ± 3.1	0.985	47.9 ± 2.9	47.7 ± 3.1	0.188
Early neonatal death, *n* (%)	41 (0.7)	1 (0.2)	0.365	2 (0.4)	1 (0.2)	1.000

Data are given as mean ± SD for continuous variables and n (%) for dichotomous variables. All P values were assessed with the use of χ^2^ or Fisher’s exact test (dichotomous variables) or and t test or Mann–Whitney U test (continuous variables).

PPOS, progestin-primed ovarian stimulation; GnRH-ant, gonadotropin-releasing hormone antagonist.

Congenital malformations were observed in 323 of 15,245 (2.1%) in the PPOS group and 27 of 1,248 (2.2%), with a non-significant difference (P = 0.916 <0.05) as **Table 3** summarizes. When categorized by deliveries and sex, no between-group comparison of birth defects was found. In addition, apart from the overall analysis of the prevalence of congenital malformations, a detailed breakdown of congenital malformations was further analyzed according to the various organ systems, which was statistically insignificant. Consistently, the reanalysis of PSM demonstrated that the incidence of congenital malformations remained insignificant.

**Table 3:** Congenital malformations of live-born infants between the PPOS and GnRH-ant groups.

	Before matching			After matching		
	PPOS(*n* = 15,245)	GnRH-ant(*n* = 1,248)	*P*-value	PPOS(*n* = 1,300)	GnRH-ant(*n* = 1,247)	*P*-value
No. of infants with malformation, *n* (%)	323 (2.1)	27 (2.2)	0.916	27 (2.1)	27 (2.2)	0.877
Category by deliveries, *n*/*N* (%)						
Singletons	190/9,389 (2.0)	14/830 (1.7)	0.506	19/776 (2.4)	14/829 (1.7)	0.284
Twins	133/5,856 (2.3)	13/418 (3.1)	0.272	8/524 (1.5)	13/418 (3.1)	0.102
Category by sex, *n*/*N* (%)						
Male	197/7,968 (2.5)	19/659 (2.9)	0.517	16/657 (2.4)	19/659 (2.9)	0.614
Female	126/7,274 (1.7)	8/588 (1.4)	0.503	10/643 (1.6)	7/587 (1.2)	0.586
No. of malformations	375	32		32	34	
Types of malformations, *n*			–			–
Nervous system (Q00-Q07)	5	2		0	3	
Eye, ear, face and neck (Q10-Q18)	33	6		4	5	
Circulatory system (Q20-Q28)	173	15		19	15	
Respiratory system (Q30-Q34)	16	2		1	3	
Cleft lip and cleft palate (Q35-Q37)	8	0		1	1	
Digestive system (Q38-Q45)	36	4		1	4	
Genital organs (Q50-Q56)	10	0		3	0	
Urinary system (Q60-Q64)	19	2		0	2	
Musculoskeletal system (Q65-Q79)	48	1		2	1	
Other malformations (Q80-Q89)	24	0		1	0	
Chromosomal anomalies (Q90-Q99)	3	0		0	0	

Data are given as n (%) for dichotomous variables. All P values were assessed with the use of the χ^2^ test.

PPOS, progestin-primed ovarian stimulation; GnRH-ant, gonadotropin-releasing hormone antagonist.

Just as **Table 4** presents, after adjusting for a variety of confounders, the results for PTB and LBT of singletons and twins could not be changed. Also, no evidently elevated risk of congenital malformations was seen in infants born after PPOS treatment in comparison with the GnRH-ant treatment in both singletons (adjusted OR 0.81, 95% CI 0.47–1.41) and twins (adjusted OR 1.32, 95% CI 0.74–2.37). The results were still invariable after reanalysis using PSM (**Table 4**).

**Table 4:** Crude and adjusted odds ratios (ORs) of neonatal outcomes in live-born singletons and twins between the PPOS and GnRH-ant groups.

	Before matching		After matching		
	Crude OR(95% CI)	Adjusted OR(95% CI)	Crude OR(95% CI)	Adjusted OR(95% CI)
**Singletons**					
Low birth weight (<2,500 g)	1.29 (0.82-2.02)	1.32 (0.84-2.07)	1.22 (0.68-2.22)	1.26 (0.69-2.30)
Preterm delivery (<37 weeks)	1.40 (0.94-2.07)	1.45 (0.97-2.15)	1.57 (0.95-2.59)	1.58 (0.95-2.64)
Congenital malformations	0.83 (0.48-1.44)	0.81 (0.47-1.41)	0.68 (0.34-1.38)	0.66 (0.32-1.34)
**Twins**					
Low birth weight (<2,500 g)	0.93 (0.76-1.13)	0.92 (0.75-1.13)	0.91 (0.70-1.18)	0.90 (0.69-1.18)
Preterm delivery (<37 weeks)	0.84 (0.69-1.02)	0.83 (0.68-1.01)	0.85 (0.66-1.10)	0.82 (0.62-1.07)
Congenital malformations	1.38 (0.78-2.46)	1.32 (0.74-2.37)	2.07 (0.85-5.04)	2.43 (0.97-6.06)

Multivariate analyses were conducted when adjusted for maternal age, maternal BMI, duration of infertility, pregnancies, parity, cause of infertility, sperm origin, fertilization method, FET endometrial preparation, endometrial thickness, number of embryos transferred, and embryo stage transferred.

OR, odds ratio; CI, confidence interval; BMI, body mass index; FET, frozen embryo transfer.

## Discussion

Recently, the PPOS protocol has been suggested to reduce the incidence of OHSS and produce similar pregnancy outcomes compared with conventional protocols. However, the safety about the novel protocol needs to be well settled. As far as we know, our study is the largest retrospective cohort study to investigate the neonatal outcomes and congenital malformations after PPOS treatment compared with GnRH-ant treatment considering and matching the possible influence factors as much as possible to date. We found that PPOS was a safe choice of ovarian stimulation for offspring without compromised neonatal outcomes or increasing the risk of congenital malformations.

In the prior decades, the effects of P on oocyte and embryo development both *in vivo* and *in vitro* remained controversial. Fukui et al. also found that adding P to the *in vitro* culture system could decrease the rate of bovine oocyte maturation (27). Moreover, a study conducted by Silva et al. showed that P could have a negative effect on blastocyst yield for *in vitro* matured bovine oocytes, which could be partially reversed by mifepristone, namely P antagonist (28). By contrast, Bezerra et al. observed that in the medium-sized bovine antral follicles, P could promote the growth of oocytes after prematuration *in vitro* (29). For rhesus monkeys, the oocyte maturation induced by P could also be observed (30). Carter et al. demonstrated that elevated P did not affect the proportion of *in vitro* fertilized embryos developing to the blastocyst stage (31). Overall, the consensus has not been reached, and the inconformity of data might be contributed to the difference in study design or the species-specific difference. Additionally, several retrospective studies suggested that the cumulative live birth rate or ongoing pregnancy rate was inversely associated with serum P concentration on the day of hCG triggering in fresh embryo transfer cycles, indicating that the serum P level could reduce endometrial receptivity (32, 33). This could be avoided with the wide popularization of FET nowadays. However, concerns about long-time exposure to P of oocytes and embryos have been raised by physicians, and relatively few studies have been involved in this field.

Kuang et al. proposed the first PPOS protocol in 2015 based on the prior study that confirmed the use of exogenous gonadotropin in the luteal phase, which was luteal-phase ovarian stimulation (LPS) (12, 34). Moreover, advanced vitrification techniques and the freeze-all embryo strategy made the protocol widely used. In some prospective and retrospective cohort studies, comparable embryological and clinical outcomes were observed between the PPOS protocol and the conventional ovarian stimulation protocol, which implied that the embryo development potential could not be impaired (35, 36). A previous study has demonstrated no significantly elevated rate of congenital anomalies for infants after the treatment with LPS compared with the conventional ovarian stimulation protocol (37). Apart from these findings, the impact of P on newborn babies has been investigated in the aspects of P supplement before and during pregnancy. Carmichael et al. found that the maternal use of oral contraceptives during early pregnancy was associated with an increased odd of hypospadias (38), whereas Zhang et al. proposed that the use of emergency contraception could not have adverse effects on pregnancy outcomes (39). Therefore, the safety of P for oocytes and embryos still needs to be examined further.

To further address this issue, a detailed analysis using a retrospective study containing a large amount of samples was run to investigate the safety for the PPOS protocol with regard to the neonatal outcomes and the occurrence of birth defects. In our study, we demonstrated that the neonatal outcomes and the incidence of congenital malformations were always comparable between the PPOS protocol and the GnRH-ant protocol after matching and adjusting for various confounding factors, suggesting that P could not have an adverse effect on the safety for offspring, which was in line with previous studies (14–17). Our study also found that the cardiovascular malformations were the most common defects at birth among all types of congenital malformations in both the PPOS and GnRH-ant groups, as studies previously reported. Furthermore, the overall incidence of congenital malformations in live-born infants was 2.1% in our present study, nearly in accordance with a data-linkage cohort study of IVF newborns in China (2.0%) (40).

The greatest strength of our study is that we analyze the data with more than 10,000 newborn samples to make the conclusion reliable. However, the minor differences were easy to be significant for the baseline characteristics due to the large sample size of the study, so we have adopted PSM analysis to balance the between-group differences of baseline characteristics, which could reduce the systematic bias. Moreover, the results were confirmed after using multivariable regression analysis controlling for vital confounding factors. In addition, the data of the study were collected in a unified way, and its analysis was performed in a single IVF center including applying for the same IVF procedures and vitrification or thawing procedures to avoid possible errors. Nevertheless, the study also has its limitations. The retrospective cohort study has its inherent defects easily leading to selection bias; therefore, a more rigorous prospective randomized controlled study is required to increase the strength of our study. Furthermore, although we have analyzed the results considering the influential factors as much as possible, there were still some unknown confounders not accounted into the study.

In conclusion, our study suggests that the PPOS protocol with a comparison of the GnRH-ant protocol is safe for offspring in terms of newborn outcomes and congenital malformations. In the near future, PPOS treatment might become an appealing option for infertile women. Moreover, the long-term follow-up for the health of babies born after treatment with PPOS is still further needed to testify the safety of this protocol.

## Data availability statement

The raw data supporting the conclusions of this article will be made available by the authors, without undue reservation.

## Ethics statement

The studies involving human participants were reviewed and approved by the institutional ethics committee of the ninth hospital. The patients/participants provided their written informed consent to participate in this study.

## Author contributions

XL, YK, and RC conceived of and designed the study. DL collected the data, performed the statistical analysis, and drafted the first version of the manuscript. ZH, QC, and WC helped making interpretation for the data. All authors contributed to the manuscript revision and read and approved the submission of the final version of the manuscript.

## Funding

This research was supported by grants from the National Natural Science Foundation of China (no.81873856), the “Two-hundred Talent” (20191814), Shanghai Health and Family Planning Commission (201940287), and the Shanghai Science and Technology Innovation Fund (22Y11906000; 22Y21900800).

## Acknowledgments

We sincerely appreciate the support and cooperation from all staff members of the Department of Assisted Reproduction in Shanghai Ninth People’s Hospital.

## Conflict of interest

The authors declare that the research was conducted in the absence of any commercial or financial relationships that could be construed as a potential conflict of interest.

## Publisher’s note

All claims expressed in this article are solely those of the authors and do not necessarily represent those of their affiliated organizations, or those of the publisher, the editors and the reviewers. Any product that may be evaluated in this article, or claim that may be made by its manufacturer, is not guaranteed or endorsed by the publisher.
