# Supplementary material for: Neonatal outcomes and congenital malformations in children born after progestin-primed ovarian stimulation protocol
Source: Front Endocrinol (Lausanne). 2022 Nov 9;13:965863. doi: 10.3389/fendo.2022.965863 (PMC9681814; doi:10.3389/fendo.2022.965863)
Supplement: Supplementary file 1 [file DataSheet_1.docx]

Supplementary Material


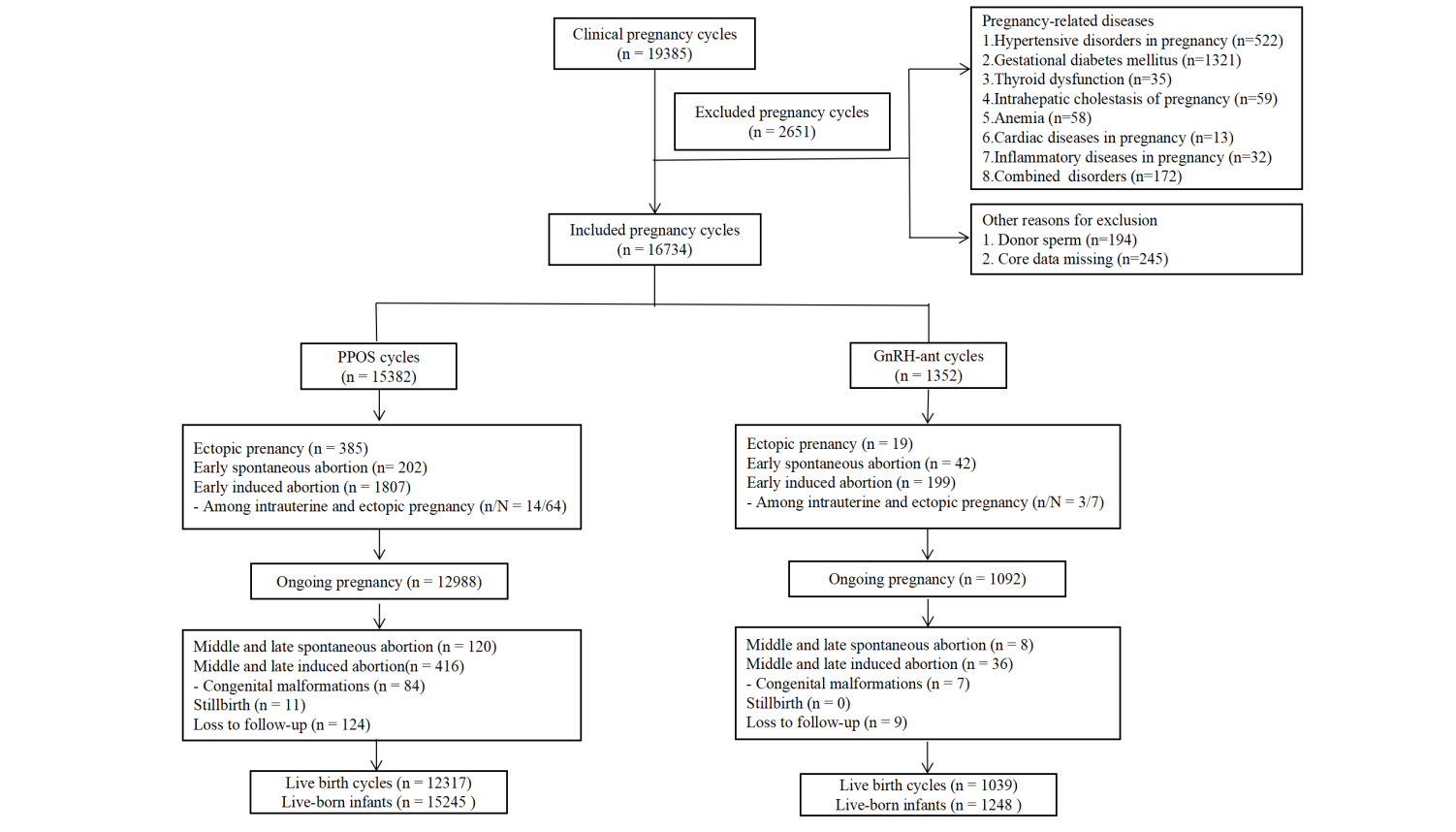


**Supplemental figure 1.** The flow diagram of the study design and cohort selection

**Supplemental table 1.** Congenital malformations of fetuses from selective terminations of pregnancy due to fetal anomalies between the PPOS and GnRH-ant groups

|  | PPOS  (*n* = 15382) | GnRH-ant  (*n* = 1352) | *P*-value |
| --- | --- | --- | --- |
| Cycles with malformations, *n* (%) | 84(0.5) | 7(0.5) | 0.892 |
| No. of malformations, *n* | 97 | 9 |  |
| Types of malformations, *n* |  |  | - |
| Nervous system (Q00-Q07) | 11 | 1 |  |
| Eye, ear, face and neck (Q10-Q18) | 2 | 1 |  |
| Circulatory system (Q20-Q28) | 18 | 0 |  |
| Respiratory system (Q30-Q34) | 2 | 0 |  |
| Cleft lip and cleft palate (Q35-Q37) | 4 | 0 |  |
| Digestive system (Q38-Q45) | 3 | 1 |  |
| Genital organs (Q50-Q56) | 0 | 0 |  |
| Urinary system (Q60-Q64) | 4 | 0 |  |
| Musculoskeletal system (Q65-Q79) | 4 | 1 |  |
| Other malformations (Q80-Q89) | 20 | 1 |  |
| Chromosomal anomalies (Q90-Q99) | 28 | 4 |  |

**Note:** Data are presented as n (%) for dichotomous variables. All P values were assessed with the use of x^2^ test or Fisher’s exact test.

Abbreviations: PPOS = Progestin-primed ovarian stimulation, GnRH-ant = Gonadotrophin releasing hormone antagonist
